# Childhood Lead Exposure and Cognitive Functioning Among Older Adults: Evidence From the Health and Retirement Study

**DOI:** 10.1093/geroni/igab046.1212

**Published:** 2021-12-17

**Authors:** Haena Lee, Mark Lee, John Robert Warren

**Affiliations:** 1 University of Southern California, Los Angeles, California, United States; 2 University of Minnesota, Minneapolis, Minnesota, United States

## Abstract

Many children born in the early 20th century were exposed to water-borne lead, a neurotoxin that negatively impacts brain development. While lead exposure has been linked to poor cognition among children and young adults, no population-level research has examined the long-term implications of lead exposure for cognitive functioning in later life. Our study is the first to address this gap by utilizing novel data linkages between the 1940 U.S. Census and the Health and Retirement Study (HRS). Our sample includes respondents who were under age 17 (born 1924-1940) by the time of the decennial enumeration on April 1, 1940. Given that the dominant source of lead exposure was water during this period, we assessed lead exposure by using water chemistry and piping material data for each HRS respondent’s city of residence in 1940. Late-life cognitive functioning for HRS participants (observed 1998-2016) was measured using the Telephone Interview for Cognitive Status. We find that lead exposure during childhood is significantly and negatively associated with cognitive functioning in later life. HRS participants who lived in cities with lead pipes and acidic or alkaline water—the conditions required for lead to leech into municipal water—showed lower levels of cognitive functioning decades later as compared to other participants. This association persisted net of race, gender, childhood socioeconomic status and childhood health. However, the association was largely accounted for by adjusting for educational attainment. This implies that childhood lead exposure impacts later-life cognition via its effect on educational attainment.

